# Data-driven refinements for voice disorder classification: improving accuracy and generalisability

**DOI:** 10.3389/fdgth.2026.1800552

**Published:** 2026-06-23

**Authors:** Rijul Gupta, Catherine Madill, Craig Jin

**Affiliations:** 1 Computing and Audio Research Laboratory, School of Electrical and Computer Engineering, The University of Sydney, Sydney, NSW, Australia; 2 Voice Research Laboratory, Sydney School of Health Sciences, Faculty of Medicine and Health, The University of Sydney, Sydney, NSW, Australia

**Keywords:** acoustic features, cross-database generalisation, data-driven classification, machine learning, multi-class classification, voice disorders

## Abstract

**Introduction:**

As machine-learning models for vocal pathology advance, their performance is increasingly constrained not by modelling techniques but by the taxonomic structures used to define the classification task itself. Conventional clinical frameworks, while grounded in diagnostic practice, often reflect conceptual groupings that do not map cleanly onto the acoustic patterns learned by modern Voice AI systems—contributing to the persistent performance gap between multi-class and binary detection tasks. Motivated by this mismatch, we introduce an alternative strategy: deriving a taxonomy from data-driven acoustic relationships rather than prescriptive clinical categories, with the goal of establishing a more model-aligned and generalisable foundation for voice disorder classification.

**Methods:**

We developed CarLab 2025, a novel data-driven classification framework derived from model confusion patterns. We conducted comprehensive experiments comparing its performance against existing clinical taxonomies, including the hierarchical USVAC 2025 framework, as well as Compton 2022, da Silva Moura 2024, and Za’im 2023, across multiple vocal tasks, features, and model architectures. We evaluated both in-domain performance and cross-database generalisation, including experiments with multi-task learning and targeted data injection.

**Results:**

CarLab 2025 achieved superior in-domain classification accuracy compared to established clinical taxonomies, with balanced accuracy reaching 67.20% compared to 61.03% for the best-performing clinical framework. For out-of-domain generalisation, models trained with structured taxonomies consistently outperformed those trained with narrow, single-disorder labels, and training on a diverse set of vocal tasks proved more effective for cross-database performance than relying on a single task. Multi-task learning offered no advantage over single-task training, and while injecting a small amount of data from target domains significantly boosted binary detection accuracy, this improvement did not consistently translate to multi-class recall.

**Discussion:**

Our experiments established a baseline performance exceeding that obtained with existing clinical classification frameworks by aligning more closely with acoustic manifestations of disorders. We further show that exposure to varied recording conditions is crucial for binary generalisation, while robust multi-class generalisation will require substantially more diverse multi-source training data. The results provide a clear, evidence-based path toward developing more robust and generalisable models for vocal pathology detection.

## 1 Introduction

Voice-based artificial intelligence (AI) is an emerging field with the potential to revolutionise healthcare by enabling non-invasive, scalable monitoring of a wide range of health conditions. One of the most promising applications is the use of voice as a biomarker [1]. However, for vocal biomarkers to be effectively leveraged in detecting systemic or neurological diseases, their ability to accurately identify and classify primary voice disorders must first be rigorously validated. Voice disorders provide a direct and pronounced manifestation of impaired vocal function, making them an ideal testbed for developing and evaluating AI-driven acoustic analysis methods. Establishing strong performance in voice disorder classification is therefore a critical step toward the broader goal of reliable, clinically meaningful vocal biomarkers [2]. Furthermore, automated, objective, and robust systems for identifying voice disorders could help alleviate persistent challenges in healthcare systems, including limited specialist availability and long diagnostic waiting times.

Despite this potential, multi-class voice disorder classification remains a significant challenge in clinical voice assessment. Although automated systems can reliably differentiate between normal and pathological voices—often achieving balanced accuracies above 90% [3]—their performance drops markedly when required to distinguish among specific disorder types. Current multi-class systems typically achieve balanced accuracies of only 50%–60% [4], highlighting a substantial performance gap that limits their clinical applicability and underscores the need for more discriminative and reliable models.

This performance disparity stems from several factors. First, existing clinical classification frameworks were developed primarily for clinical documentation and case management, not for machine learning applications. These frameworks typically organise disorders based on case history, presumed etiology, and clinical presentation—factors that may not align with how acoustic features manifest in voice signals [5]. Second, the acoustic representations captured by signal processing techniques may differ substantially from the auditory-perceptual features that clinicians rely upon in practice, creating a mismatch between clinical taxonomies and machine-learnable patterns.

Underlying this mismatch is the source-filter view of voice production (21, 22). The vocal folds generate a quasi-periodic glottal signal whose fundamental frequency, intensity, and spectral richness depend on vocal-fold mass, tension, and adduction, and the vocal tract shapes this signal through articulator-dependent resonances. Clinical taxonomies group disorders partly by physiological mechanism and partly by management pathway (typically non-surgical vs surgical), but a model trained on the audio signal only sees the integrated source-filter output.

Hierarchical clinical classification frameworks [6] can potentially improve multi-class classification performance compared to narrow, disorder-specific labels; however, these frameworks remain grounded in clinical taxonomies that may not optimally reflect acoustic similarities as captured by machine learning models [4]. We hypothesise that classification accuracy might be substantially improved by deriving groupings directly from model-based acoustic feature similarities, rather than from pre-existing clinical taxonomies.

The choice of voice sample also shapes what physiological information is available to the model. The sustained vowel /a/, produced with a relatively unobstructed vocal tract, foregrounds glottal-source behaviour and is the standard task for measuring jitter, shimmer, and harmonic-to-noise ratio. The sustained vowels /i/ and /u/ introduce articulator-driven changes in formant structure and effective vocal-tract length, exposing source-filter interaction. Connected speech adds co-articulation, prosody, and the patient’s own compensatory adaptations, but its voiceless segments contribute little source-driven information and can dilute pathological cues if not handled carefully [5]. Our experiments train models across all four task types to take advantage of this complementarity.

Beyond in-domain performance, a critical challenge also lies in model generalisation across different databases and recording environments. Models trained on one dataset often fail to maintain performance when evaluated on data from different institutions, recording setups, or populations. This limitation severely constrains the practical deployment of automated classification systems in diverse clinical settings.

This study addresses three key research questions: (1) Can a data-driven classification framework, derived from model confusion patterns, achieve superior classification accuracy compared to existing, clinically-defined taxonomies on an in-domain dataset? (2) To what extent does the choice of classification framework, model architecture, and vocal task affect a model’s ability to generalise to unseen, out-of-domain databases? (3) Can strategic interventions, such as multi-task learning or targeted data injection, mitigate performance degradation in cross-database classification tasks?

Our key contributions are: (1) We present CarLab 2025, a novel data-driven classification framework that demonstrably outperforms existing taxonomies on in-domain multi-class classification. (2) We provide a comparative analysis of cross-database performance, revealing that structured, clinically-informed taxonomies (including CarLab 2025) offer greater robustness than narrow, single-disorder labels. (3) We demonstrate that while multi-task architectures offer no significant benefit, performance on out-of-domain data is improved by training on diverse vocal tasks and augmenting the training set with even a small number of samples from different recording environments.

## 2 Methods

We describe our experimental setup, including data selection, the derivation of the CarLab 2025 classification framework, model architectures, and training procedures.

### 2.1 Data selection

For same-database training, validation, and testing, we used a subset of the Saarbruecken Voice Database (SVD) [7]. We targeted diagnostic categories with at least 50 unique patients so that the subsequent 70/10/20 stratified split by patient would in expectation place at least 10 distinct patients in the test split. We then applied a strict post-split exclusion: any class with fewer than 10 distinct patients in the realised test split was dropped, since per-class recall computed over fewer than 10 patients is not statistically meaningful. The 9 surviving classes and their per-split, per-sex patient counts are given in Table 1; two of these (Leukoplakia and Vocal Fold Polyp) fell below the 50-patient target after data quality filtering but were retained because their realised test splits still exceeded the 10-patient floor.

**Table 1:** SVD same-database demographics: per-class patient counts in each split, shown as female/male.

Class	Train	Val	Test
Normal	235/195	51/12	86/41
Functional Dysphonia	39/28	11/1	16/5
Hyperfunctional Dysphonia	41/79	16/0	35/1
Laryngitis	41/29	1/6	10/4
Leukoplakia	12/7	4/2	8/2
Psychogenic Dysphonia	39/11	7/0	12/2
Recurrent Paralysis	68/44	14/2	25/10
Reinke’s Edema	25/7	6/0	17/0
Vocal Fold Polyp	12/16	0/3	5/6

Splitting is patient-level: every audio session for a given patient appears in exactly one split. Test counts (column 3) all meet the 10-patient floor; Leukoplakia and Vocal Fold Polyp fell below the 50-patient pool target after data quality filtering and were retained because their test splits still cleared the floor.

Splitting was strictly patient-level: every audio session for a given patient appears in exactly one of the splits, so there is no speaker leakage. A session corresponds to a single recording visit and contains multiple audio clips, one per elicitation task: the sustained vowels /a/, /i/, and /u/, and the German phrase *Guten Morgen, wie geht es Ihnen?*. We did not remove voiceless segments from the phrase, since the connected speech as elicited is what a deployed system would receive at inference. All audio was resampled to 16 kHz mono. MFCCDD was computed with 25 ms windows and 10 ms hop, with per-clip mean-variance normalisation; the SSL features were used with their native windowing and without further normalisation. At evaluation time, frame-level softmax probabilities were averaged within a clip, and clip-level probabilities were averaged within a session, giving one prediction per session.

For cross-database evaluation, we used four different databases: The Advanced Voice Function Assessment Databases (AVFAD) [8], Massachusetts Eye and Ear Infirmary (MEEI) [9], Uncommon Voice [10], and VOICED (23). We initially considered TORGO [11] as a fifth source, but its diagnostic labels (dysarthria subtypes) do not overlap with any of the disorders represented in the SVD training pool or in the clinical taxonomies under comparison, so it cannot be mapped into our label space and was not used in any reported experiment. We extracted all the data from the four retained databases and used it solely as a test set, applying the same preprocessing pipeline. The details of the data used from these cross databases are provided in Table 2.

**Table 2:** Demographics of data for cross dataset experiments presented as diagnosis ((female/male) count).

Dataset	Demographics
AVFAD	Normal (2749/1243), Acute Laryngitis (22/22), Amyotrophic Lateral Sclerosis (33/11), Bilateral Recurrent Laryngeal Nerve Paralysis Peripheral (22/22), Laryngeal Mucosa Trauma Chemical and Thermal (77/33), Leukoplakia (33/11), Muscle Tension (55/22), Muscle Tension Adaptive (0/44), Non intubation related vocal fold granuloma (44/22), Parkinsons (0/33), Presbyphonia (77/44), Puberphonia (0/11), Reactive Vocal Fold Lesion (11/0), Reinke’s Edema (241/88), Unilateral or Bilateral recurrent laryngeal nerve paresis (22/11), Unilateral Recurrent Laryngeal Nerve Paralysis (264/22), Varix and Ectasia of Vocal Fold (88/55), Ventricular Dysphonia (33/33), Vocal Fold Cyst Sub-Epithelial (220/33), Vocal Fold Hemorrhage (77/22), Vocal Fold Nodules (286/22), Vocal Fold Polyp (220/77), Vocal Fold Scar (121/22), Vocal Fold Sulcus (11/11)
MEEI	Normal (60/36), Abductor Spasmodic Dysphonia (2/0), Adductor Spasmodic Dysphonia (14/0), Conversion Dysphonia (8/4), Cyst (0/2), Episodic Functional Dysphonia (2/0), Exudative Hyperkeratotic Lesions of Epithelium (0/2), Hemmoragic Reinke’s Edema (0/2), Hyperfunction (46/28), Laryngeal Tuberculosis (0/2), Leukoplakia (0/4), Paralysis (12/9), Paresis (2/4), Polypoid Degeneration Reinke’s (4/0), Post Intubation Submucosal Edema Mild (2/0), Presbyphonia (4/2), Scarring (2/0), Subglottal Mass (0/2), Subglottal Stenosis (0/2), Varix (0/4), Vocal Fold Edema (4/0), Vocal Fold Nodules (8/2), Vocal Fold Polyp (6/8), Vocal Tremor (2/0)
Uncommon Voice	Normal (484/296), Pathologial (2292/621)
VOICED	Normal (36/21), Hyperkinetic Dysphonia (28/15), Hyperkinetic Dysphonia Nodule (3/2), Hyperkinetic Dysphonia Polyps (3/3), Hyperkinetic Dysphonia Presbyphonia (0/1), Hyperkinetic Dysphonia Reinke’s Edema (2/1), Hyperkinetic Dysphonia Rigid Vocal Fold (0/1), Hyperkinetic Dysphonia Spasmodic Dysphonia (1/0), Hyperkinetic Dysphonia Vocal Fold Nodules (1/0), Hyperkinetic Dysphonia Vocal Fold Paralysis (1/0), Hypokinetic Dysphonia (19/3), Hypokinetic Dysphonia Adduction Deficit (0/1), Hypokinetic Dysphonia Adduction Deficit (1/0), Hypokinetic Dysphonia Conversion Dysphonia (1/0), Hypokinetic Dysphonia Glottic Insufficiency (4/3), Hypokinetic Dysphonia Vocal Fold Paralysis (4/0), Reflux Laryngitis (19/19)

Furthermore, for database augmentation experiments, we prepared two new datasets that contained all of the data from the same-database experiments, with additional data injected as follows: (1) One male and one female normal speaker, plus one male and one female speaker with any pathology (selected randomly from available pathologies) from each of the databases MEEI, Uncommon Voice, and VOICED. (2) One male and one female normal speaker only from each of the three databases MEEI, Uncommon Voice, and VOICED. We refer to these regimes as *inj. P/N* and *inj. N* respectively. Each injection adds only two speakers per class per source database, so the absolute volume of injected data is small relative to the SVD training pool; this design probes whether limited exposure to a target domain’s recording conditions is enough to alter generalisation, rather than whether large-scale multi-domain training is.

### 2.2 Deriving a diagnosis map from model confusion—CarLab 2025

**Figure 1:**
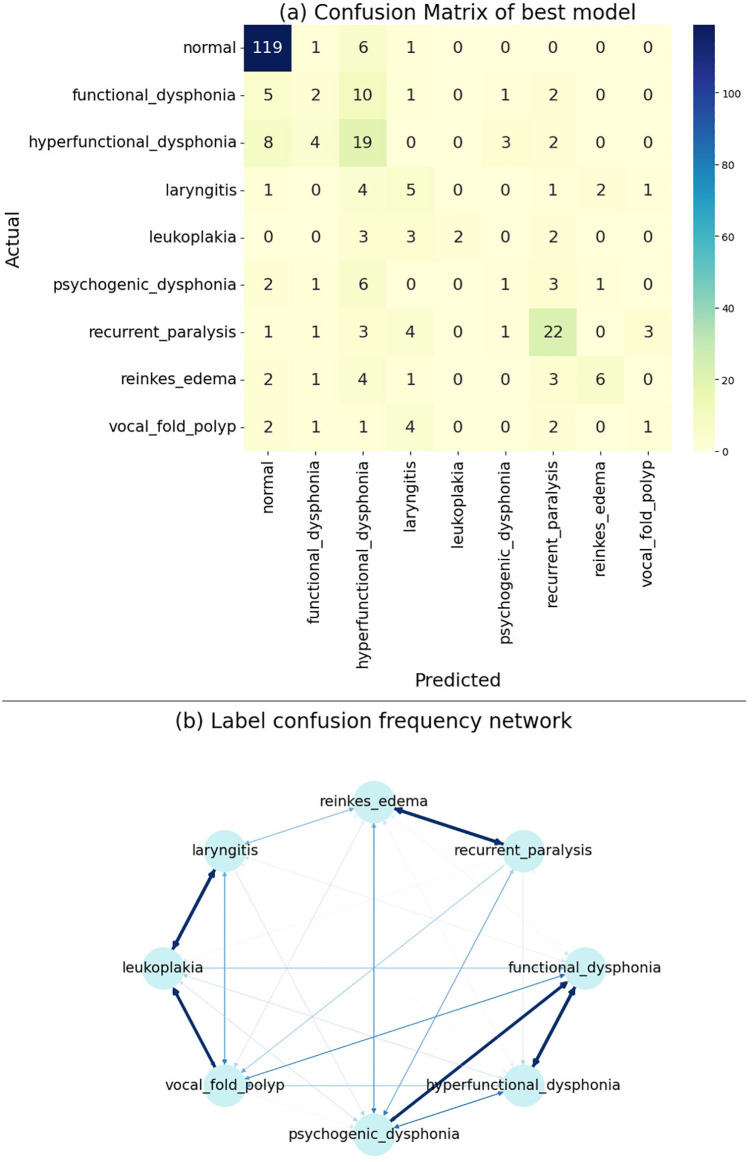
Derivation of CarLab 2025 framework from model confusion. **(a)** Confusion matrix of model with best classification performance on narrow classification task. **(b)** Network representation of classes to merge that result in maximum classification accuracy improvement.

We derived a new diagnosis map (CarLab 2025) automatically based on the model’s classification performance on narrow classifications. We first trained a model on the raw input classes without using any predefined classification framework. In this scenario, the model attempts to classify each diagnosis directly, but inevitably it cannot achieve perfect classification. The resulting confusion matrix for this model is shown in Figure 1a.

The procedure to derive the hierarchy from this confusion matrix has two stages. In the first stage we use the predictions of the narrow-classification model (trained on the 9 individual classes) as input to a greedy merge search; the search itself does not train any new model. Let y and $\hat{y}$ denote the actual and predicted labels produced by the narrow model on its test split, and let the active set L be the unique pathological labels (Normal is held out and never merged). At each step we evaluate every unordered pair (a,b)∈L by relabeling all occurrences of a and b in y and $\hat{y}$ to a common placeholder and computing the resulting balanced accuracy; the pair that maximises balanced accuracy is committed to the hierarchy. The two committed labels are then replaced by the placeholder in y and $\hat{y}$, the active set is reduced by one, and the search continues until L contains a single super-pathological class. Ties are broken by the iteration order over unordered pairs, which is deterministic. The full merge trajectory is retained, so any intermediate level of granularity can be recovered by reading the trajectory at the desired depth. Pseudocode for this stage is given in Algorithm 1. In the second stage, once the hierarchy is fixed, we train fresh models directly on the merged labels at each level of granularity (Auto A/B/C plus Normal at the level reported in Table 3). The numbers in Table 3 are produced by these stage-two models, which are independent of the narrow stage-one model.

Algorithm 1 **Greedy post-hoc merge search for CarLab 2025.**

1: **Input:** actualy, predicted $\hat{y}$ from a trained model

2: L←unique(y)∖{Normal}                ▹ active pathological labels

3: H←[]               ▹ ordered hierarchy of merges

4: **for** k=0,1,…,|L|−1 **do**

5:  best←−∞

6:  **for** each unordered pair (a,b)∈L **do**

7:   $y^{\prime },{\hat{y}}^{\prime }\leftarrow$copies of $y,\hat{y}$ with a,b replaced by placeholder k

8:   $acc\leftarrow balanced\_accuracy(y^{\prime },{\hat{y}}^{\prime })$

9:   **if** acc>best **then**

10:    best←acc; $\left(a^{*},b^{*})\leftarrow (a,b\right)$

11:   **end** **if**

12:  **end** **for**

13:  append $\left(a^{*},b^{*},best\right)$ to H

14:  replace $a^{*},b^{*}$ in $y,\hat{y}$ with placeholder k

15:  $L\leftarrow (L\setminus \{a^{*},b^{*}\})\cup \{k\}$

16: **end** **for**

17: **return** H

**Table 3:** Balanced accuracy comparison of CarLab 2025 and other frameworks with in-domain testing.

Framework	Balanced accuracy
CaRLab 2025	**67.20**
USVAC 2025 Level 1	61.03
Compton 2022	56.61
da Silva Moura 2024	63.09
Za’im 2023	56.60

Bold values mark the best-performing configuration.

The CarLab labels themselves are chosen, by Algorithm 1, to match the confusion structure that the stage-one narrow model produced on SVD training set; stage-two models are trained on these labels and evaluated on a held out test set derived from the same SVD database. The stage-two model parameters are not reused from stage one, but there is overlap in the underlying database. The four cross-database evaluations in Section 3.3 are fully external to both stages and provide the more decisive generalisation test.

Once the hierarchy is obtained, we map the existing databases with this new hierarchical classification system using the same systematic process used for other clinical classification frameworks. This mapping process transforms the original diagnostic labels present in each database (e.g., “Vocal Fold Polyp,” “Reinke’s Edema”) into the categorical labels defined by the CarLab 2025 framework (e.g., “Auto A,” “Auto B,” “Auto C”). The mapping is facilitated using the diagnostic mapping toolkit (divr-diagnosis^1^ ), an open-source Python module that enables systematic organisation and querying of clinical voice disorder diagnoses using hierarchical taxonomies. The toolkit implements hierarchical taxonomies as weighted directed acyclic graphs, allowing flexible querying and projection of diagnostic labels across different levels of granularity. For each diagnostic label in the database, we identify which classes in the CarLab 2025 hierarchy it belongs to based on the merging decisions made during hierarchy construction. Once a diagnostic label is correctly identified and mapped to the hierarchy, we can easily retrieve the diagnosis at any level of the classification hierarchy.

We ran the merge search independently for models trained on three feature sets (MFCCDD, Wav2Vec, UnispeechSAT) crossed with five input configurations (connected speech, the sustained vowels /a/, /i/, and /u/, and the union of all four tasks), giving 15 hierarchies in total. To aggregate across them, for every pair of original pathological labels we measured the merge distance in each hierarchy as a proxy for the depth of their lowest common ancestor: pairs merged early have small distance, pairs that meet only at the root have large distance. We normalised these distances by the maximum distance in each hierarchy and averaged across the 15 hierarchies. Figure 1b renders the resulting graph: nodes are the original labels, and edge thickness encodes the inverse of the aggregated distance, so thicker edges connect labels that were routinely merged early. We highlight, for each node, the edge to its nearest neighbour under this metric; this reveals three distinct groups that emerge consistently across the feature and task sweep. Following only these first preferences avoids further overlap between groups, which would otherwise lead to mode collapse into binary or ternary classifications. We label these three groups as Auto A, Auto B, and Auto C. Auto A consists primarily of Functional Dysphonia, Hyperfunctional Dysphonia, and Psychogenic Dysphonia. Auto B includes Laryngitis, Leukoplakia, and Vocal Fold Polyp. Auto C groups Reinke’s Edema and Recurrent Paralysis together. The constituent classes of these labels are detailed in Figure 1b as the nodes with the thickest edges.

### 2.3 Features, models, and training

We used MFCC with delta and double deltas (MFCCDD), Wav2Vec [12], and UnispeechSAT [13], as these performed best in previous work on voice disorder detection [14]. UnispeechSAT and Wav2Vec are calculated using the S3PRL library [15], and MFCCDD using Pytorch-Audio [16].

Additionally, we trained a model using a combination of all connected speech and vowels /a/, /i/, and /u/. This model has the opportunity to learn patterns across different vocal tasks, which may improve performance either due to the increased data available for training or because the model can learn patterns that are not specific to connected speech or any particular vowel, but are present across different vocalisation tasks.

The designed model processes all frames of UnispeechSAT, Wav2Vec, or MFCCDD for each audio session. It uses a deep feedforward network as a readout layer, consisting of two stacks of Linear, ReLU, and LayerNorm with a hidden size of 1024. The model was initialised with orthogonal weights before training. The output of the model generates a classification vector using softmax for each frame. These frame-level probabilities are averaged to compute the average probability for an audio clip, and the probabilities from all audio clips within a session are further averaged to obtain the session-level probability.

In addition to the standard training routine, where we average the probabilities of each audio clip to calculate the probability per session and use that as the loss function, we also train models using a multi-audio loss approach. In this setting, each audio clip within a session contributes independently to the loss, and the total loss is computed by aggregating the clip-level losses across the session. This encourages the model to produce consistent predictions across all clips rather than relying on a single dominant sample. During evaluation, clip-level probabilities are again averaged to obtain a session-level prediction. This approach effectively mimics an ensemble across multiple audio clips, while requiring only a single trained model rather than multiple independently trained models. Additionally, the same model learns patterns across different vocal tasks, namely vowel /a/ and connected speech, which were the best performing tasks in our preliminary experiments.

Models based on Wav2Vec and UnispeechSAT are trained for 200 epochs, whereas those using MFCCDD are trained for 2,000 epochs. This discrepancy arises because Wav2Vec and UnispeechSAT are pretrained models, whereas MFCCDD, not inherently specialised for this task, requires additional training time to converge. All models are trained with a learning rate of $10^{-6}$, using the Adam optimiser [17] and Cross Entropy Loss, alongside class weights to address sample size variation across the classes. For model evaluation, we use balanced accuracy. All experiments were conducted with fixed random seeds to ensure reproducibility, and results are reported as mean and standard deviation across five independent runs.

### 2.4 Multi-task experiments

**Figure 2:**
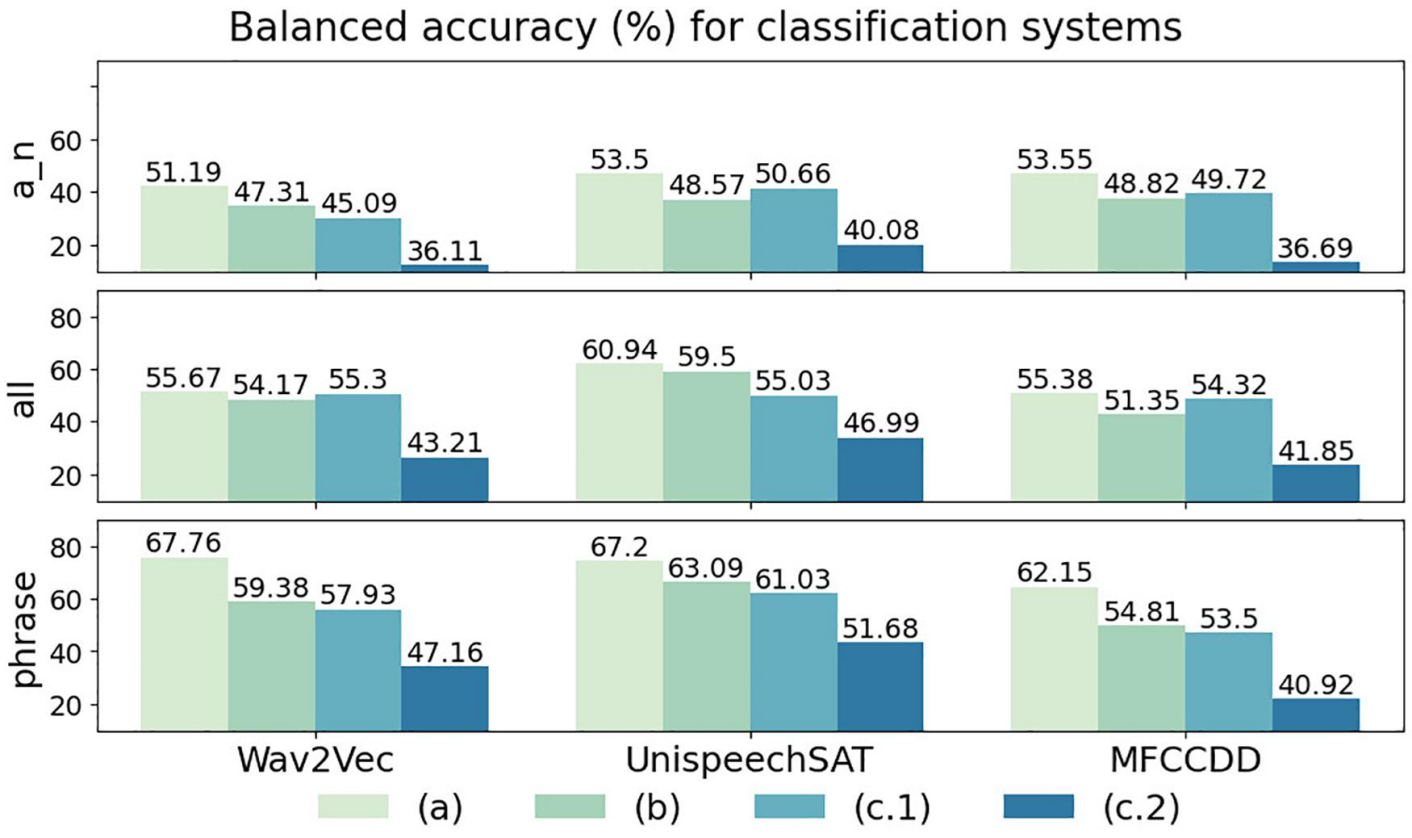
Multi-task and multi-criterion design.

In our multi-task experiments, we trained models with multiple readout layers—one for each diagnostic level of interest. We also evaluated a multi-criterion model, which shares the same core architecture as the single-task model but differs in how outputs are handled. Instead of producing predictions at multiple levels directly, it outputs only at the most granular diagnostic level. Macro categories are then derived using the diagnostic mapping toolkit (divr-diagnosis^2^ ), an open-source Python module that facilitates working with multiple clinical classification frameworks by enabling systematic organisation and querying of clinical voice disorder diagnoses using hierarchical taxonomies. Figure 2 illustrates this distinction in architecture and supervision: the multi-task model learns to predict at each level simultaneously, whereas the multi-criterion model predicts only at the most specific level, with broader categories inferred post hoc.

For the multi-task model, each diagnostic level produces an independent output, and a separate cross-entropy loss is computed for each. These losses are summed to form the final training objective. In contrast, the multi-criterion model generates class probabilities at the most narrow level of classification (N). These predictions are then projected to broader levels (n<N) using the diagnostic hierarchy. Each projected output is evaluated using its own cross-entropy loss, and the resulting losses are similarly aggregated. To mitigate the effects of data imbalance, class weights are applied in both multi-task and multi-criterion training.

### 2.5 Cross-database testing

To evaluate the generalisability of our models, we conducted extensive out-of-domain, cross-database testing across all experimental configurations. Owing to limitations in data availability and quality, the SVD database was used as the sole training source. As discussed in Section 2.1, SVD was the only dataset that simultaneously provided sufficient overlap with different clinical classification frameworks, high-fidelity audio recordings, and a broad range of elicitation tasks, along with diagnostic labels compatible with the classification schemes required for robust multi-class classification.

The datasets used for cross-database evaluation are summarised in Table 2. For each experiment, we deployed models previously trained on SVD and evaluated their performance on unseen databases without any retraining or fine-tuning.

An exception was made for the data-injection experiments, in which we trained models using SVD augmented with varying proportions of data from MEEI, VOICED, and Uncommon Voice. These models were then evaluated not only on the injected datasets themselves but also on AVFAD, a fully held-out database. This design allowed us to assess both direct transferability and the potential of selective injection to improve cross-domain robustness.

## 3 Results

We evaluated the performance of CarLab 2025 against existing clinical classification frameworks, assessed its generalisability across different databases, and explored several strategies to improve cross-database robustness. Our experiments demonstrate that the data-driven framework achieves superior in-domain classification accuracy while revealing significant challenges in out-of-domain generalisation. The following sections present these findings in detail.

### 3.1 Performance of CarLab 2025

**Figure 3:**
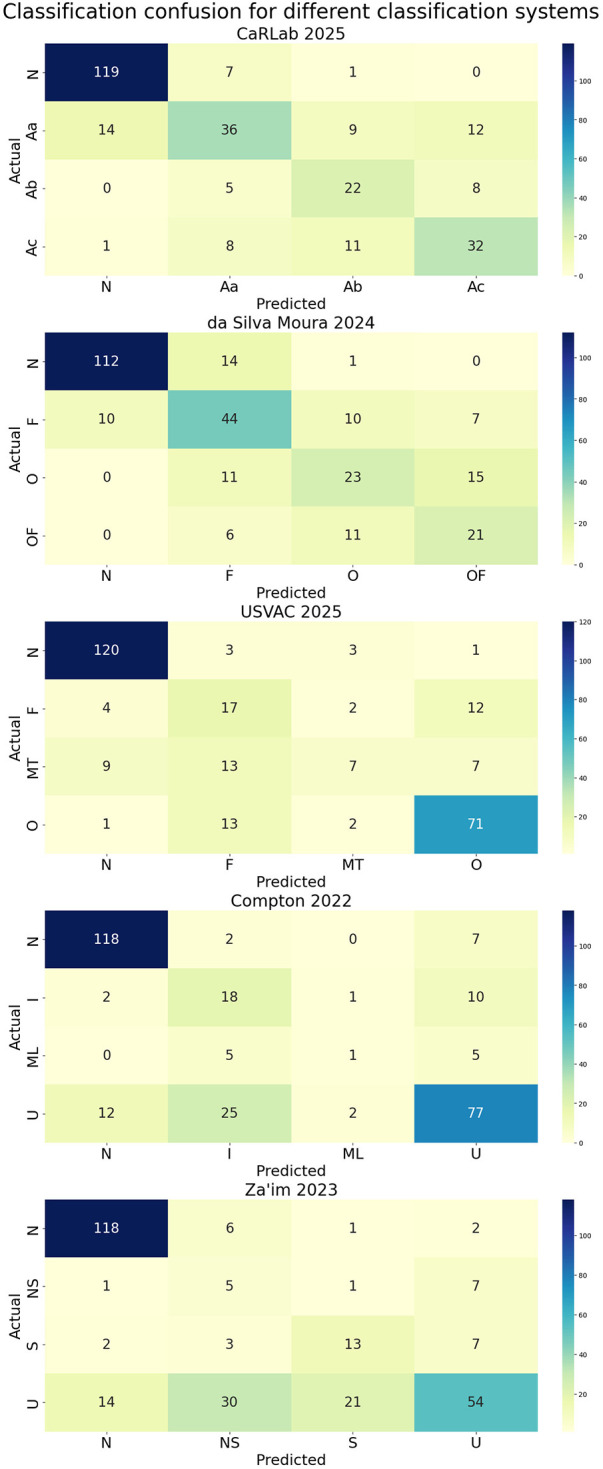
Balanced accuracy comparison of CarLab 2025 and other frameworks with in-domain testing. (a), (b), (c.1), and (c.2) represent the three clinical classification frameworks CarLab 2025, da Silva Moura 2024, and Levels 1 and 2 of USVAC 2025. It is evident that our new clinical classification framework CarLab 2025 results in ML models that outperform the rest of the frameworks in every category.

**Figure 4:**
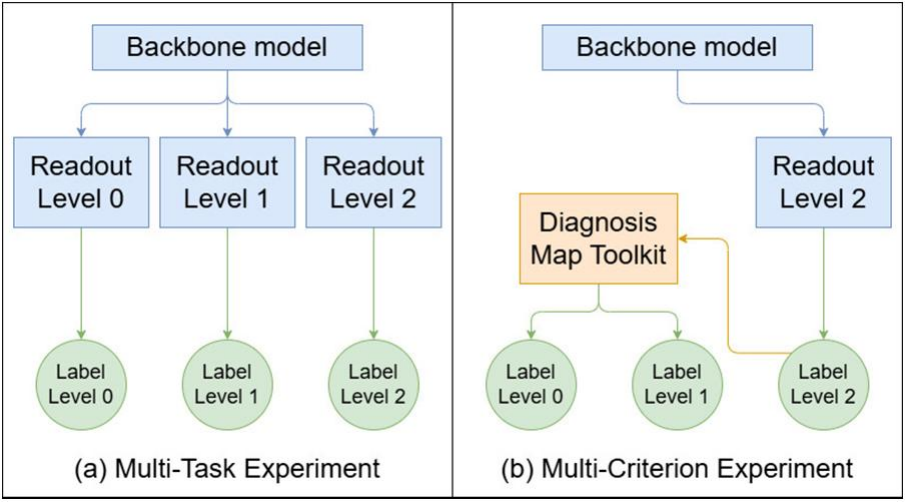
Confusion matrices for classification using different clinical classification frameworks. First level classification using different clinical classification frameworks in decreasing order of balanced classification accuracy. Multiple pathologies collapse into Organic for USVAC 2025 Level 1, and Unallocated for Compton 2022 and Za’im 2023 which severely limits their clinical utility. Keys for diagnosis labels: {Aa, Auto A; Ab, Auto B; Ac, Auto C; F, Functional; I, Inflammatory; MT, Muscle Tension; ML, Mass Lesions; N, Normal; NS, Non Structural Dysphonia; O, Organic; OF, Organofunctional; S, Structural Dysphonia; U, Unallocated}.

We compared the balanced accuracy of models trained using our proposed CarLab 2025 framework against those trained with existing clinical taxonomies: USVAC 2025 (a hierarchical clinical classification framework [6], tested at Levels 1 and 2), Compton 2022 [18], da Silva Moura 2024 [19], and Za’im 2023 [20]. As shown in Table 3, CarLab 2025 achieved a balanced accuracy of 67.20%, outperforming all other frameworks, with the next best being da Silva Moura 2024 at 63.09% and USVAC 2025 Level 1 at 61.03%. When we compared performance across different input tasks and features, as shown in Figure 3, our new classification framework consistently resulted in ML models that outperformed those trained using other frameworks, with performance differences ranging from 0.37% to 8.38%. The confusion matrices for all classification frameworks are presented in Figure 4, where the diagonal for CarLab 2025 is brighter than those of other frameworks, indicating uniform performance improvement across all classes.

To quantify the strength of these comparisons we use a paired Wilcoxon signed-rank test together with a paired bootstrap percentile 95% confidence interval on the mean difference. The Wilcoxon test is a non-parametric paired test that ranks the absolute paired differences between two methods and asks whether the signs of those ranks are consistent with the null hypothesis of zero median difference; we use the exact null distribution rather than the normal approximation since our sample sizes are small. The bootstrap interval reports the effect size on the same paired data, which for small n is more directly informative than a p-value alone. There are two natural paired samples already present in the manuscript: the 9 (feature, task) cells of Figure 3, which give one paired balanced-accuracy difference per cell between CarLab 2025 and each reference taxonomy, and the 9 underlying-class rows of Table 4, which give one paired recall difference per disorder. Table 5 reports the outcomes for both samples; we additionally cross-checked them against a sign test, a paired t-test, and a sign-flip permutation test, all of which give the same conclusions on every comparison.

**Table 4:** Per Class Average recall for CarLab 2025 and other frameworks with in-domain testing (%).

Label	CarLab 2025	Narrow	da Silva Moura 2024	USVAC 2025 Level 1	USVAC 2025 Level 2
Functional Dysphonia	51±20	7±6	47±18	39±16	28±22
Hyperfunctional Dysphonia	54±8	37±13	54±12	28±13	36±8
Laryngitis	60±14	38±10	25±15	63±18	47±17
Leukoplakia	58±14	6±7	44±22	71±23	16±12
Normal	75±16	81±12	83±7	84±9	78±10
Psychogenic Dysphonia	43±14	17±10	40±11	34±9	28±16
Reinke’s Edema	45±8	25±12	43±19	63±15	27±14
Recurrent Paralysis	55±11	50±19	48±17	65±16	46±12
Vocal Fold Polyp	54±12	10±8	40±15	68±9	19±14
Average	55±13	30±11	47±15	57±14	36±14

Here narrow classification includes only the disorders listed in this table.

**Table 5:** Paired statistical comparison of CarLab 2025 against each reference taxonomy. Inputs are taken directly from Figure 3 (cell-level) and Table 4 (per-class).

Comparison	n	+/n	Mean (sd) diff (pp)	95% CI (pp)	Wilcoxon p
Cell-level (Figure 3, balanced accuracy across 9 (feature, task) cells)					
CarLab 2025 vs. da Silva Moura 2024	9	9/9	+4.48(2.30)	[+3.08,+5.93]	0.004
CarLab 2025 vs. USVAC 2025 Level 1	9	9/9	+4.97(3.22)	[+2.99,+6.95]	0.004
CarLab 2025 vs. USVAC 2025 Level 2	9	9/9	+15.85(3.15)	[+14.05,+17.91]	0.004
Per-underlying-class (Table 4, recall across 9 disorders)					
CarLab 2025 vs. Narrow	9	8/9	+24.89 (19.08)	[+13.11,+36.44]	0.012
CarLab 2025 vs. da Silva Moura 2024	9	7/9	+7.89 (12.24)	[+1.11,+16.11]	0.068
CarLab 2025 vs. USVAC 2025 Level 1	9	3/9	−2.22 (14.73)	[−10.67,+7.33]	0.496
CarLab 2025 vs. USVAC 2025 Level 2	9	8/9	+18.89 (13.41)	[+10.78,+27.33]	0.008

“pp” = percentage points; “+/n” is the count of paired comparisons in which CarLab 2025 had the higher value. Mean (sd) and 95% CI are computed on the paired differences (CarLab 2025 minus reference); the CI is a paired bootstrap percentile interval over $2\times 10^{5}$ resamples. The Wilcoxon p is the two-sided exact p-value of the paired signed-rank statistic under its discrete null distribution; the per-class CarLab 2025 vs. da Silva Moura 2024 comparison contains one tied pair which the Wilcoxon test drops before computing W. We additionally verified each row against a sign test, a paired t-test, and a sign-flip permutation test, all of which agreed with the Wilcoxon conclusions.

At the framework level, CarLab 2025 has higher balanced accuracy than da Silva Moura 2024, USVAC 2025 Level 1, and USVAC 2025 Level 2 in all 9 cells of Figure 3; the bootstrap 95% CI on the mean per-cell difference is strictly positive in every case ([+3.08,+5.93], [+2.99,+6.95], and [+14.05,+17.91] percentage points respectively) and the exact Wilcoxon p-value reaches its smallest possible value at this sample size, $2/2^{9}\approx 0.004$. Compton 2022 and Za’im 2023 are not present in Figure 3 at the per-cell level but lie 10.59 and 10.60 percentage points below CarLab 2025 in Table 3 respectively. The picture is more nuanced at the per-underlying-class level of Table 4: CarLab 2025 is significantly higher than USVAC 2025 Level 2 (p≈0.008, CI [+10.78,+27.33]) and the narrow taxonomy (p≈0.012, CI [+13.11,+36.44]), borderline-significant against da Silva Moura 2024 (p≈0.068, CI [+1.11,+16.11] excluding zero), and not significantly higher than USVAC 2025 Level 1, which is on average 3 percentage points higher per class (CI [−10.67,+7.33] spanning zero). The framework-level balanced-accuracy advantage of CarLab 2025 therefore does not come from uniform per-class superiority; it comes from stronger performance on the classes that other taxonomies confuse most. The more decisive evidence for the framework remains the cross-database analysis in Section 3.3, which is external to CarLab’s derivation.

To understand why CarLab 2025 outperformed other frameworks, we calculated recall per underlying class—defined as the proportion of true instances of a class that are correctly identified—and compared the resulting deltas between CarLab 2025 and the other frameworks. The results in Table 4 show that da Silva Moura 2024 generally underperforms relative to CarLab 2025, with almost all classes exhibiting lower average recall, despite better performance in the “normal” class. This improvement is insufficient to offset the poor performance in other classes.

USVAC 2025 Level 1 presents a more complex scenario. While several individual labels in USVAC 2025 Level 1 outperform those in CarLab 2025 (e.g., Leukoplakia achieves 71% recall in USVAC 2025 Level 1 vs. 58% in CarLab 2025), the overall system performance of models trained with USVAC 2025 Level 1 is lower than those trained with CarLab 2025. This is because the classes labelled as Functional Dysphonia, Hyperfunctional Dysphonia, and Muscle Tension Dysphonia in USVAC 2025 Level 1 perform poorly compared to when these same classes are categorised within CarLab 2025’s Auto A class. Since balanced accuracy weighs each class equally, the overall performance suffers despite some classes in USVAC 2025 Level 1 significantly outperforming CarLab 2025. When comparing per-class recall, USVAC 2025 Level 1 only outperforms CarLab 2025 by 3% on average, and the range of performance overlaps significantly. At Level 2, CarLab 2025 outperforms USVAC 2025 by 19% on average recall, with no overlap in performance.

Merging classes where USVAC 2025 Level 1 outperforms could potentially improve CarLab 2025’s performance, but this would lead to mode collapse, resulting in ternary comparisons, which is undesirable.

### 3.2 Multi-task experiments

**Figure 5:**
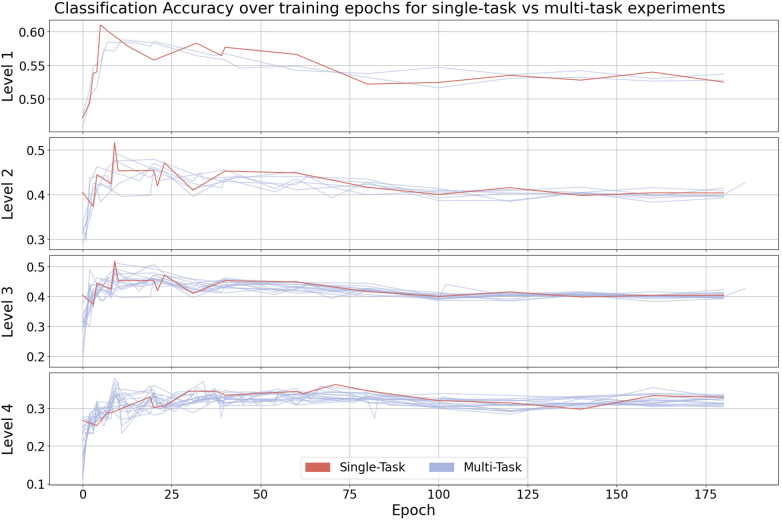
Multi-task classification accuracy with in-domain testing. Balanced classification accuracy of models trained on multiple levels of classification hierarchy (multi-task) converge to similar level as those trained on a single level.

We investigated whether multi-task or multi-criterion training could improve performance by providing the model with hierarchical label information simultaneously, potentially leading to a more robust feature representation and higher accuracy. As shown in Figure 5, models trained to classify a diagnosis at multiple levels simultaneously, either as multi-task (separate classification for levels 0 to n) or as multi-criterion (classification at level n hierarchically mapped to levels 0 to n−1), all converge to similar classification accuracy at level n.

Furthermore, as shown in Table 6, there is no consistent improvement or reduction in classification accuracy when training in a multi-task or multi-criterion regime. Treating the 5 classification levels in Table 6 as a paired sample, a paired Wilcoxon signed-rank test fails to reject the null of zero median difference between Single-Task and Multi-Task (p=0.19, mean diff +2.30 pp in favour of Single-Task, 95% bootstrap CI [−0.13,+4.10] spanning zero) or between Single-Task and Multi-Criterion (p=1.00, mean diff +0.22 pp, CI [−1.95,+2.51]). We hypothesise that incorporating multiple levels of the clinical classification hierarchy does not meaningfully increase the diversity of the training signal, given the overlap in classes, which may explain the limited gains observed in these experiments. Since these setups complicate training significantly without providing clear benefits, we do not recommend this approach. Training on the most detailed level of classification is sufficient, as broad categorisation levels can always be obtained by mapping the corresponding classes using the diagnosis maps.

**Table 6:** Balanced accuracy (%) on multiple levels of classification for UnispeechSAT trained with phrase with different regimes with in-domain testing.

Level	Single-task	Multi-task	Multi-criterion
0	90.20	**92.57**	90.46
1	**58.01**	55.82	53.61
2	47.17	42.59	**49.54**
3	47.17	42.90	**49.54**
4	**36.23**	33.42	34.51

Bold values indicate the highest entry within each row.

### 3.3 Performance on cross-database evaluation

**Figure 6:**
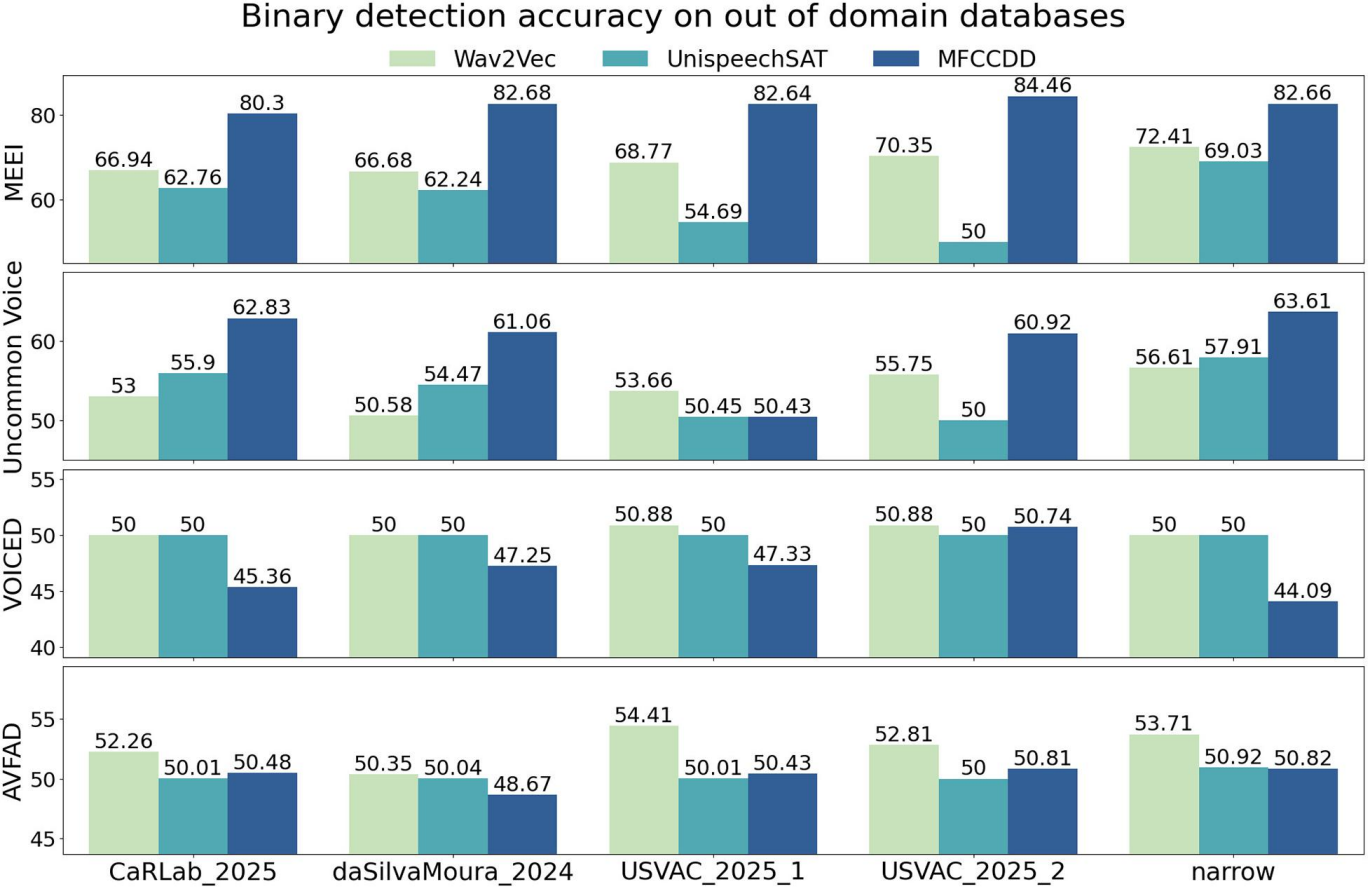
Binary detection accuracy on cross-database evaluation. Balanced binary detection accuracy for different out of domain databases shows that most models (trained with SVD) achieve approximately the random chance accuracy of 50%. Surprisingly MFCCDD performs better here than the NN feature based models when it manages to out perform random chance. Note: y-axis ranges differ across the four database panels (e.g., MEEI clusters in 60%–85% while AVFAD clusters tightly around the 50% chance line), so a shared axis would compress within-database structure; values above each bar permit precise comparison.

We evaluated how models trained solely on the SVD dataset performed on four unseen databases to assess out-of-domain generalisation. Since SVD is the only training source, we cannot fully separate “the framework generalises” from “the framework generalises from SVD”; we therefore treat this analysis as exploratory rather than confirmatory. With this caveat in mind, it still quantifies the magnitude of domain shift and compares how different classification frameworks withstand it before any interventions are applied. As shown in Figure 6, the baseline performance reveals a significant degradation, with most models struggling even on binary detection tasks. This suggests that the models have likely become too attuned to the source database. This is further supported by the fact that MFCCDD performs better when models achieve more than 50% accuracy (indicating complete mode collapse to a single classification label). Despite being trained for ten times more epochs in our setting, this feature set is significantly less trained than the pretrained Wav2Vec and UnispeechSAT models.

Due to the limited annotation granularity in Uncommon Voice—restricted to binary normal/pathological labels—no further stratified analysis is feasible. VOICED, on the other hand, exhibited complete mode collapse in our models, with nearly all samples misclassified as normal, rendering it uninformative for evaluating fine-grained classification. In contrast, MEEI yielded considerably more informative results. However, it must be noted that MEEI’s recording setup differs systematically between normal and pathological samples, introducing potential confounds in binary detection tasks.

**Figure 7:**
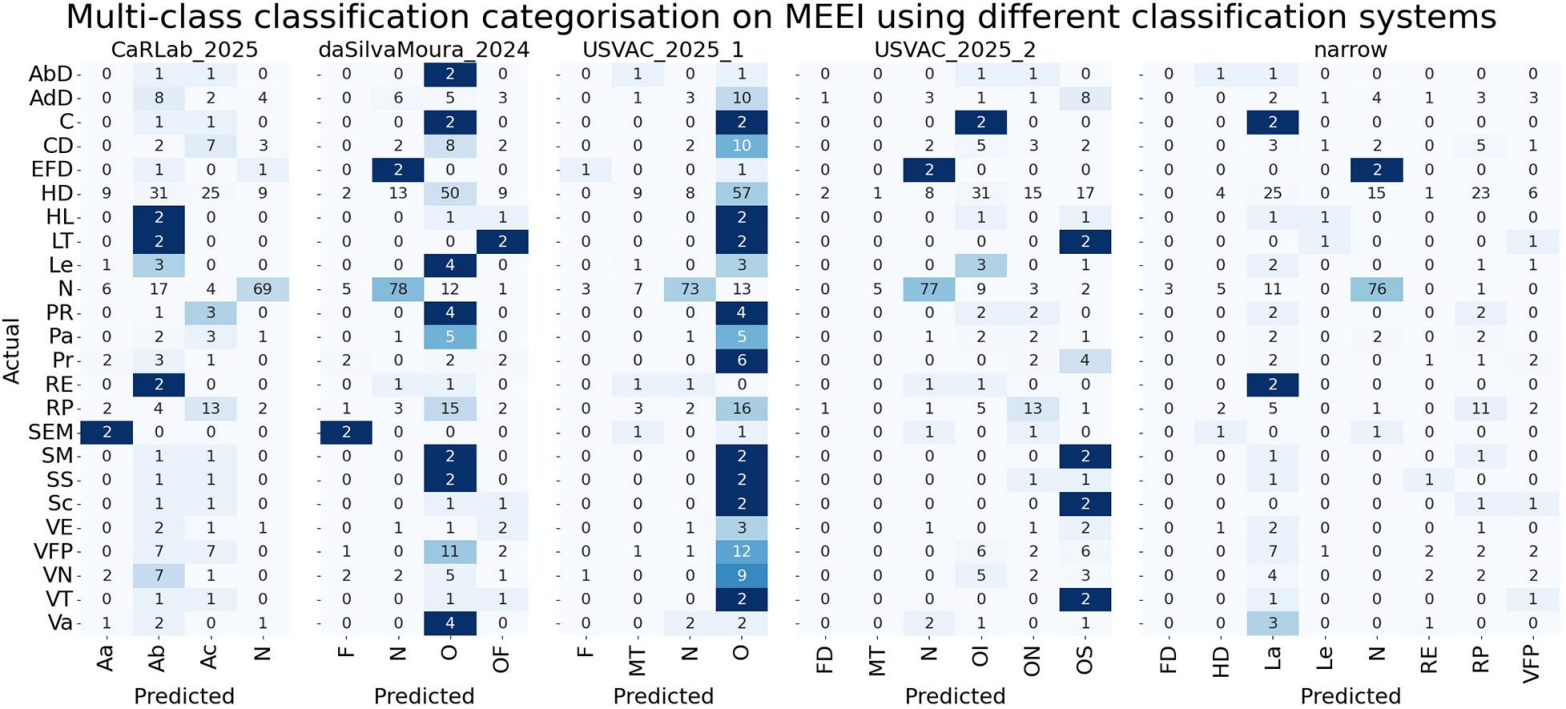
Classification of MEEI pathology labels using models trained on various clinical taxonomy schemes. Although the narrow classification approach achieved the best binary detection performance, it underperforms in the more clinically relevant task of differentiating among pathological classes, relative to systems trained using structured clinical taxonomies. Keys for diagnosis labels: {Aa, Auto A; Ab, Auto B; Ac, Auto C; AbD, Abductor Spasmodic Dysphonia; AdD, Adductor Spasmodic Dysphonia; C, Cyst; CD, Conversion Dysphonia; EFD, Episodic Functional Dysphonia; F, Functional; FD, Functional Dysphonia; HD, Hyperfunction/Hyperfunctional Dysphonia; HL, Exudative Hyperkeratotic Lesions Of Epithelium; I, Inflammatory; La, Laryngitis; Le, Leukoplakia; LT, Laryngeal Tuberculosis; ML, Mass Lesions; MT, Muscle Tension; N, Normal; NS, Non Structural Dysphonia; O, Organic; OF, Organofunctional; OI, Organic Inflammatory; OII, Organic Inflammatory Infective; ON, Organic Neuro Muscular; ONP, Organic Neuro Muscular Peripheral Nervous Disorder; OS, Organic Structural; OSE, Organic Structural Epithelial Propria; P, Pathological; Pa, Paresis; PD, Psychogenic Dysphonia; PR, Polypoid Degeneration Reinke’s; Pr, Presbyphonia; RE, Hemmoragic Reinke’s Edema/Reinke’s Edema; RE, Reinke’s Edema; RP, Recurrent Paralysis/Paralysis; S, Structural Dysphonia; Sc, Scarring; SEM, Post Intubation Submucosal Edema Mild; SM, Subglottal Mass; SS, Subglottis Stenosis; U, Unclassified; Va, Varix; VE, Vocal Fold Edema; VFP, Vocal Fold Polyp; VN, Vocal Fold Nodules; VT, Vocal Tremor}.

Despite this limitation, MEEI remains highly valuable for studying inter-pathology discrimination. Pathological samples within MEEI were recorded under comparable acoustic and procedural conditions, allowing us to isolate model performance on intra-pathological differentiation. In Figure 7, we evaluate how the best-performing binary detection models from Figure 6 classify the MEEI dataset when trained using different clinical taxonomies. Since the diagnostic categories present in MEEI are not aligned with the CarLab 2025 framework or da Silva Moura 2024 framework, we treat this as a form of open-set classification: we map true diagnostic labels from MEEI to predicted categories produced by each classification system.

To emphasise class affinities, we normalise the rows of each confusion matrix and apply a fourth-power transformation, amplifying stronger associations and suppressing background noise. This visualisation strategy enables clearer identification of emergent patterns.

Three observations emerge from this analysis: (1) The model trained with USVAC Level 1 framework exhibits near-total mode collapse, assigning the majority of samples to only two categories—*Normal* and *Organic*—irrespective of true pathology. This undermines its utility for any meaningful downstream diagnostic application. (2) Across all systems, only a small subset of MEEI labels demonstrate strong affinity to predicted categories. This is not unexpected, given the limited diagnostic diversity in our training set, which included only nine unique disorders. (3) Notably, the model trained using the narrow taxonomy—which previously achieved the highest binary detection accuracy—performs worst in mapping MEEI diagnoses to consistent categories. It exhibits fewer strong affinities between source and predicted classes than any system trained with clinical taxonomies.

Given that several pathologies in MEEI overlap with labels used in our training data, we further examined classification performance for this subset using per-class recall, as shown in Table 7. This analysis reinforces the broader conclusion: clinical taxonomies—by grouping related disorders under shared functional or structural criteria—enable better generalisation to unseen datasets. In contrast, models trained with narrow taxonomies that treat each disorder as a distinct class offer little robustness, with minor inter-dataset deviations leading to complete misclassification. These findings support the adoption of structured, clinically meaningful taxonomies as a means of improving out-of-domain diagnostic performance.

**Table 7:** Cross database recall evaluation on MEEI using best detection model.

Label	Narrow	CarLab 2025	da Silva Moura 2024	USVAC 2025 Level 1	USVAC 2025 Level 2
Normal	79.17	71.88	81.25	76.04	80.21
Hyperfunctional Dysphonia/Hyperfunction	5.41	12.16	2.7	12.16	1.35
Leukoplakia	0.0	75.0	0.0	75.0	25.0
Reinke’s Edema/Hemmoragic Reinke’s Edema	0.0	0.0	0.0	0.0	0.0
Recurrent Paralysis/Paralysis	52.38	61.9	71.43	76.19	61.9
Vocal Fold Polyp	14.29	50.0	14.29	85.71	42.86
Average	25.21	45.16	28.28	54.18	35.22

Per-class recall (%) on MEEI using the best-performing detection model trained on SVD. Values reflect out-of-domain performance across multiple taxonomy configurations. Color intensity corresponds to relative performance, with darker shades indicating higher recall. Here narrow classification model was trained with the disorders listed in Table 4.

While the table might suggest that USVAC 2025 Level 1 yields the best results, this comparison is not on equal terms. At this level, the taxonomy reduces to three very coarse classes (Normal, Muscle Tension, and Organic) with nearly all pathology falling under a single “Organic” label. The model is therefore effectively performing a binary or ternary classification task, and its higher recall reflects a coarser problem rather than greater discriminative power. The coarse classes themselves remain clinically useful as a triage signal, but what is undermined here is the interclass variability the broader taxonomy is meant to capture; the model is no longer distinguishing between clinically distinct pathologies, which is the more demanding problem. A fairer comparison restricts attention to taxonomies that attempt fine-grained discrimination at comparable granularity: CarLab 2025 (4-class), USVAC 2025 Level 2 (5-class), and da Silva Moura 2024 (4-class). On this matched-granularity footing, the average per-class recall on MEEI in Table 7 is 45.16% for CarLab 2025, 35.22% for USVAC 2025 Level 2, and 28.28% for da Silva Moura 2024, with the narrow (single-disorder) taxonomy at 25.21%. CarLab 2025 therefore retains a clear advantage of 9.94 to 16.88 percentage points over the other matched-granularity frameworks, which is independent of any granularity-induced inflation.

By contrast, CarLab 2025 and USVAC 2025 Level 2 maintain finer-grained distinctions between pathologies while still reflecting meaningful clinical structure. CarLab 2025 includes classes distributed across three axes of dysfunction (Auto A, B, C), while USVAC 2025 Level 2 distinguishes between Muscle Tension, Organic Neuromuscular, and Organic Structural disorders. These frameworks support more realistic classification challenges and better reflect how voice disorders present in clinical settings.

Even so, performance remains far from ideal. Several pathologies receive zero recall under one or more taxonomies (Table 7), and the failures are not concentrated in any single disorder; instead, they reflect recording- and population-level shifts between SVD and MEEI that affect multiple classes with partially overlapping acoustic signatures. Consequently, changes in recording conditions, typical of cross-database evaluation, lead to a sharp drop in recall. While average recall across all classes remains above chance, the drop in accuracy relative to in-domain performance is substantial for every taxonomy except USVAC 2025 Level 1, which benefits from its coarse granularity rather than from better discriminative power.

Even under the best-performing structured taxonomy—CarLab 2025—multi-class classification suffers an additional 10% degradation when evaluated out-of-domain. These findings highlight the fragility of current models under distribution shift and the need for targeted strategies to enhance cross-database robustness, which we explore in the following sections.

### 3.4 Vocal task variations

**Figure 8:**
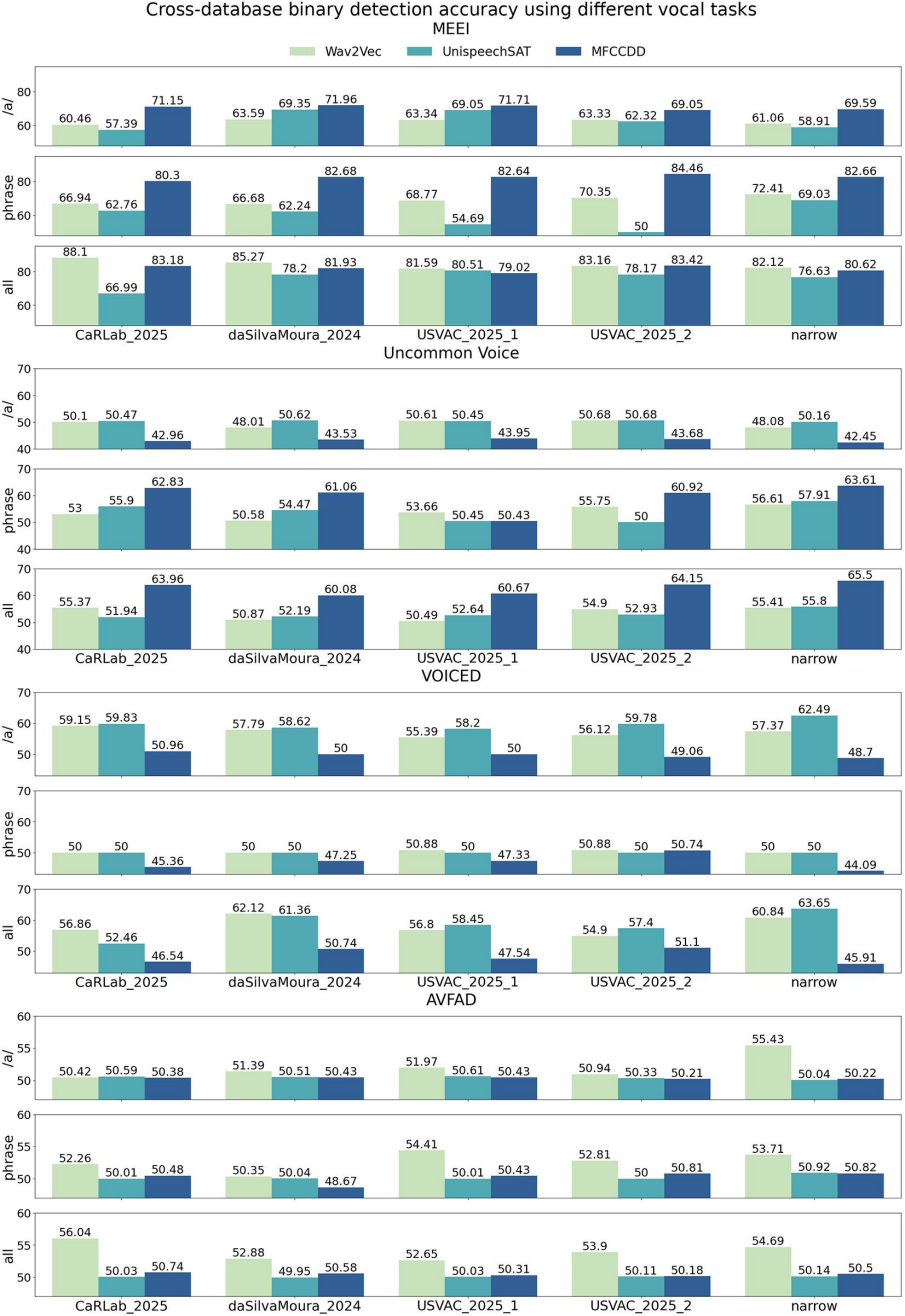
Binary detection accuracy on different databases using different vocal tasks of models trained with SVD. Within each database (MEEI, Uncommon Voice, VOICED, AVFAD) the three vocal-task rows (/a/, phrase, all) share a common y-axis to support task comparison; y-axis ranges differ across databases because absolute accuracy ranges differ. Values above each bar permit precise comparison across rows.

We examined how the choice of vocal task impacted performance across different databases. The results in Figure 8 show that models trained on a diverse combination of vocal tasks (phrase, /a/, /i/, and /u/) generalise better on cross-database binary detection tasks than models trained on a single task across four different databases: AVFAD, MEEI, VOICED, and Uncommon Voice. We observed that vowel /a/ indeed performs better on VOICED, where we only have vowel /a/ data, compared to models trained with just phrases. However, models trained with all data still outperform those trained with just /a/, indicating that the models are potentially learning features that generalise better when provided with different vocalisation tasks rather than just a single vocal task. Unfortunately, the performance of models trained with all vocal tasks on same-database testing is worse than that of models trained with phrases alone, as shown in Figure 3. This highlights the risk of falling into a performance minimum when scaling beyond a single database: if we had restricted our search to models trained with phrases alone (which performed better than models trained with all data on same-database testing), we would not have identified that using all data instead of just phrases performs better on out-of-domain testing.

### 3.5 Ensembling

Ensembling refers to the combination of predictions from multiple models or inputs to produce a single, more robust prediction, often improving generalisation and stability compared to individual models. In this work, we explore ensembling across different vocal tasks.

Given our earlier finding that vowel /a/ consistently outperforms other vowels in classification tasks—and noting that VOICED contains only vowel /a/—we conducted additional experiments focused on this vocal task. Specifically, we compared models trained on all available vocal tasks (phrase, /a/, /i/, and /u/) to models trained using only vowel /a/ and phrase.

Two configurations were tested: (1) a multi-task model where the average of the classification probabilities across /a/ and phrase was used as the loss function during training (avg), and (2) an ensemble model in which separate networks were trained on /a/ and phrase individually and then combined at inference time (ensemble).

**Figure 9:**
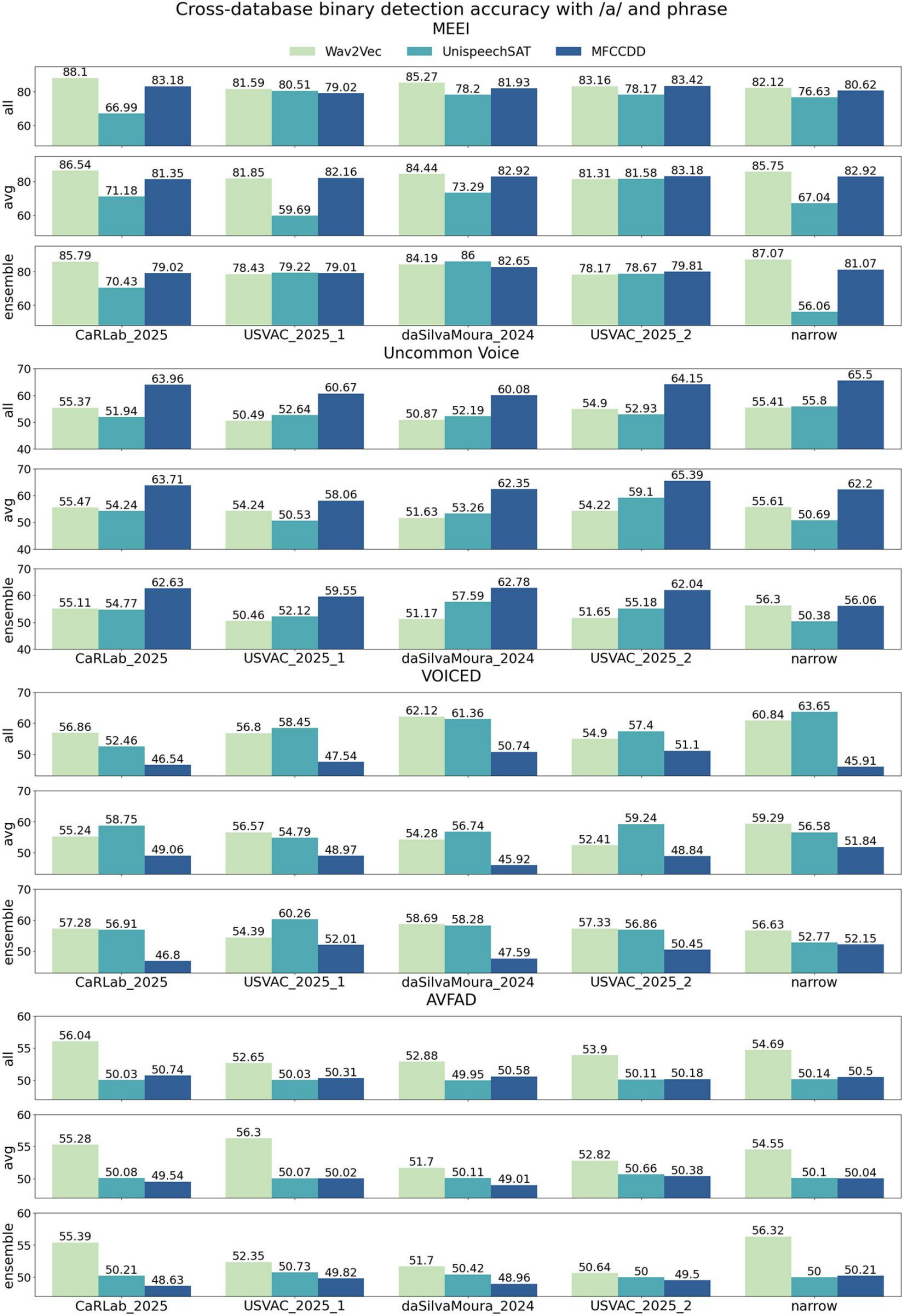
Comparison of binary detection performance across different databases using models trained on SVD. Results are shown for models trained on all vocal tasks versus those trained only on vowel /a/ and phrase. Within each database the three regime rows (all, avg, ensemble) share a common y-axis; y-axis ranges differ across databases for the same reason as Figure 8.

As shown in Figure 9, restricting training to vowel /a/ and phrase produced performance comparable to using the full set of vocal tasks, regardless of whether the tasks were combined during training (avg) or ensembled at inference. This suggests that vowel /i/ and /u/ contribute little to overall classification accuracy. These results reinforce our prior analyses, which found that vowel /a/ alone performs on par with, or better than, combinations of other vowels in binary detection tasks.

### 3.6 Data injection

Given the poor cross-database performance observed in Section 3.3, we tested whether targeted data injection could mitigate these issues. Our hypothesis was that exposing the model to the recording conditions of target domains, even with limited data, would help it learn more generalisable, pathology-specific features.

**Figure 10:**
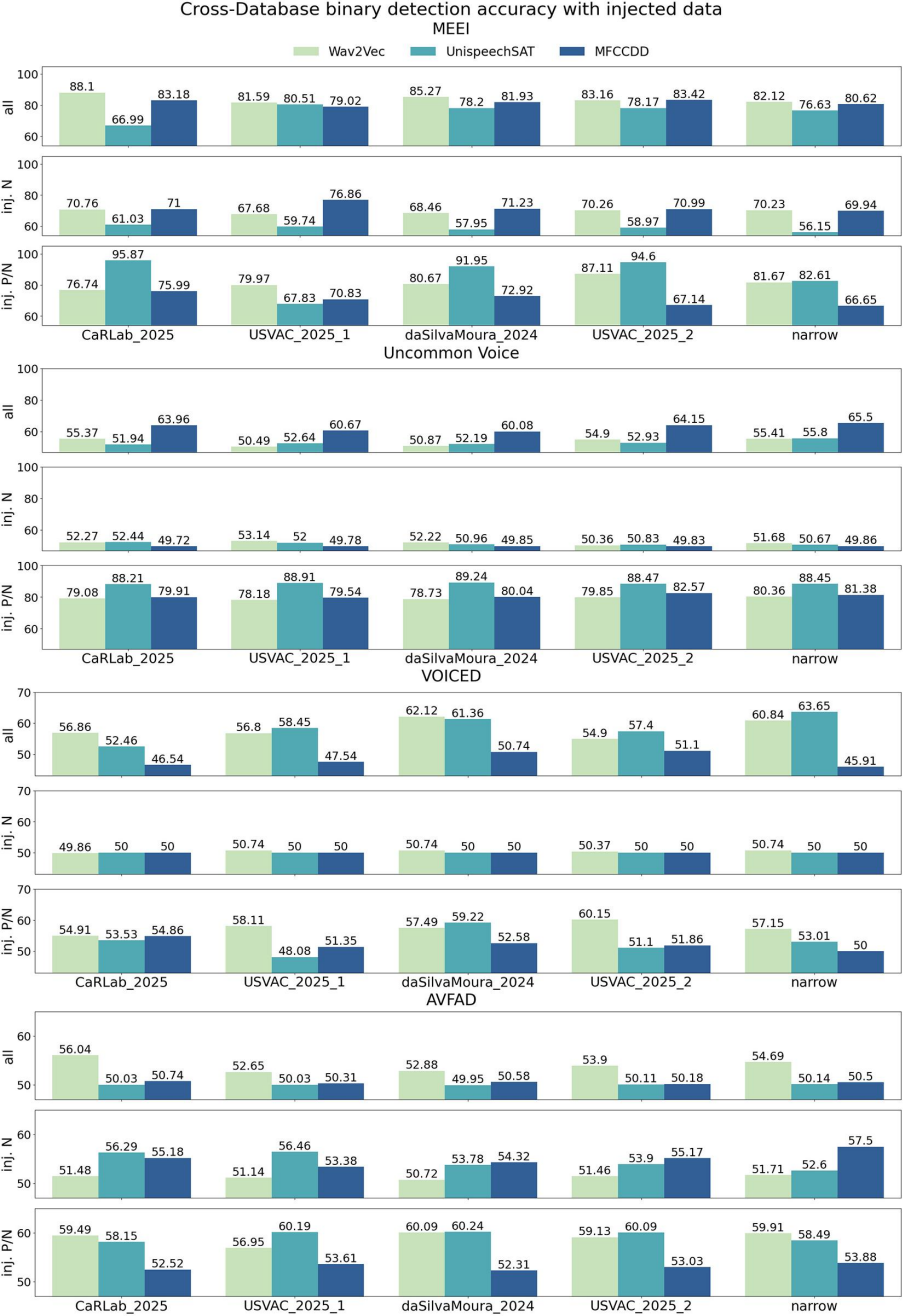
Impact of data injection on binary detection accuracy. Results on MEEI, Uncommon Voice, and VOICED are considered in-domain, as data from these sources was injected during training. In contrast, results on AVFAD constitute true cross-database testing, as this dataset was entirely held out during training. Within each database the three regime rows (all, inj. N, inj. P/N) share a common y-axis; y-axis ranges differ across databases for the same reason as Figure 8.

As shown in Figure 10, we observed an interesting result: if we inject normal data only from any particular database, the accuracy on the particular databases used for injection (MEEI, VOICED, and Uncommon Voice) drops significantly, corresponding to a mode collapse on normal only for that particular database. This supports the hypothesis that models are overfitting to the recording conditions of input data, despite the expectation that SSL-based features should be somewhat robust against this. However, even more interestingly, the performance on AVFAD, which was not used for injection, also improves, despite only normal data being injected. This indicates that the model is indeed learning about different recording conditions and can generalise this knowledge to unseen databases.

When we train with both pathological and normal samples, the model learns to perform binary detection on the other databases significantly better as well. This result is expected and confirms the benefit of data injection for improving cross-database classification performance.

### 3.7 Multi-class classification performance improvement

While the preceding experiments showed that vocal task diversity and data injection could improve binary detection, we also evaluated whether these interventions translated to meaningful gains in cross-database multi-class recall, which represents a more clinically relevant scenario. The results, however, show that these improvements do not consistently carry over. As shown in Table 8, the models trained with injected data—either pathological and normal (inj. P/N) or normal only (inj. N)—underperform models trained without injection in all except one instance (da Silva Moura 2024 on MEEI).

**Table 8:** Cross database recalls for best performing models trained with different frameworks and SVD database.

Framework	(Feature) task	Hyperfunction	Laryngitis	Leukoplakia	Normal	Reinke’s Edema	Paralysis	Polyp	Average
AVFAD									
CaRLab 2025	(MFCCDD) /a/	–	66	–	2	52	71	49	**48**
	(MFCCDD) inj. P/N	–	66	–	13	39	44	50	**42**
USVAC 2025 2	(Wav2Vec) ensemble	–	59	–	2	33	32	58	37
	(MFCCDD) inj. P/N	–	55	–	14	12	48	14	29
da Silva Moura 2024	(UnispeechSAT) phrase	–	100	–	0	3	99	5	41
	(MFCCDD) inj. P/N	–	70	–	21	12	73	11	37
narrow	(UnispeechSAT) ensemble	–	86	–	0	1	51	0	28
	(MFCCDD) inj. N	–	50	–	32	5	48	2	27
MEEI									
CaRLab 2025	(MFCCDD) /a/	4	–	25	49	50	62	86	46
	(MFCCDD) inj. P/N	7	–	100	78	0	48	64	50
USVAC 2025 2	(MFCCDD) /a/	9	–	75	44	50	43	64	48
	(Wav2Vec) inj. P/N	41	–	0	91	50	24	36	40
da Silva Moura 2024	(MFCCDD) all	3	–	75	84	50	29	86	**54**
	(UnispeechSAT) inj. P/N	16	–	100	86	100	0	71	**62**
narrow	(UnispeechSAT) /a/	11	–	0	25	50	76	0	27
	(UnispeechSAT) inj. P/N	64	–	0	73	0	0	36	29
VOICED									
CaRLab 2025	(MFCCDD) phrase	–	61	–	2	67	20	67	**43**
	(Wav2Vec) inj. P/N	–	47	–	56	0	0	33	27
USVAC 2025 2	(Wav2Vec) /a/phrase	–	21	–	30	67	20	17	31
	(Wav2Vec) inj. P/N	–	0	–	75	0	20	0	19
da Silva Moura 2024	(UnispeechSAT) phrase	–	100	–	0	0	100	0	40
	(Wav2Vec) inj. P/N	–	0	–	79	33	20	33	**33**
narrow	(MFCCDD) inj. P/N	–	0	–	0	0	0	83	17
	(UnispeechSAT) /a/	–	0	–	54	33	80	0	**33**

Bold values mark the best-performing configuration.

While models trained using CarLab 2025 continue to show strong performance on testing across both AVFAD and VOICED databases, achieving average recalls of 48% and 43% respectively, da Silva Moura 2024 performs better on MEEI (54% without injection, 62% with injection). This is largely attributed to better performance in classifying normal and assistance from injected data, as MEEI data is seen during training. Without injected data, recalls for four out of six classes are either similar or better classified by CarLab 2025, but Leukoplakia and Normal are significantly worse than those performed by da Silva Moura 2024, resulting in a net loss. We restrict comparisons to USVAC level two instead of using both levels one and two as we showed in Section 3.3 that most data under cross-database testing fell into ”Organic” class for USVAC level one and effectively results in mode-collapse.

Overall, the results do not show a clearly best-performing input task or feature. Despite our extensive suite of experiments, with all results showing better-than-chance performance, these results suggest that even more comprehensive testing is required to identify techniques that truly scale to good performance in cross-database multi-class classification. Notably, while CarLab 2025 showed mixed performance on binary detection in cross-database settings, it outperforms other classification frameworks when evaluated on multi-class classification tasks.

The most important negative finding here is that the binary-detection gains from data injection do not transfer to multi-class recall. Injection lifts binary balanced accuracy on the injected databases (Section 3.6), but the multi-class recalls in Table 8 are, in nearly every row, no better than the corresponding non-injected baseline. Treating the 12 (framework, database) average-recall pairs in Table 8 as a paired sample, a paired Wilcoxon signed-rank test on no-injection vs. P/N-injection multi-class recall fails to reject the null of zero median difference (p=0.30,W=25); the mean per-pair difference is +2.67 pp in favour of *no* injection with a 95% bootstrap CI of [−2.42,+7.33] that spans zero, so injection has no statistically detectable improvement on multi-class recall and trends mildly negative. A plausible explanation is that two speakers per class per source database is enough to expose the SSL features to a new recording channel, which is what the binary task ultimately depends on, but not enough to teach the classifier the within-pathology acoustic variability needed to discriminate disorders in an unseen domain. The binary improvement and the multi-class non-improvement should therefore be treated as separate findings, and any future work reporting data-injection gains on binary detection should report the multi-class consequences alongside.

It is also useful to disentangle the contributions of taxonomy, feature extractor, vocal task, and dataset mismatch to cross-database performance. With feature and task held fixed, structured taxonomies outperform narrow labels on multi-class recall but only marginally beat each other on binary detection, indicating that most of the cross-database binary signal is shared across taxonomies. Feature extractor is a distinct effect: in Figure 6 the SSL features (Wav2Vec, UnispeechSAT) collapse to chance accuracy on several out-of-domain databases while MFCCDD retains above-chance performance, so the feature choice can dominate the taxonomy choice in extreme distribution shift. Holding taxonomy and feature fixed, training across all four task types beats single-task training on binary detection in 4 of 4 databases (Figure 8), and this effect is largely orthogonal to taxonomy choice. The dominant residual is the dataset-mismatch cost from using SVD as the sole training source; data injection partly addresses this for the binary task and fails to address it for the multi-class task. Taxonomy choice, feature choice, and task diversity therefore contribute robustly but modestly to generalisation, while closing the dataset-mismatch cost will require substantially more diverse multi-source training data.

## 4 Discussion

### 4.1 Clinical implications of data-driven groupings

Our primary hypothesis was that a data-driven classification framework would outperform traditional taxonomies. The success of CarLab 2025 validates this hypothesis, yielding superior in-domain classification accuracy (67.20% balanced accuracy vs. 61.03% for the best clinical framework). Its data-driven groupings of vocal pathologies often diverged from traditional clinician-defined categories, yet aligned with meaningful acoustic symptomatology. For instance, clinicians at the University of Sydney Voice Clinic classified Hyperfunctional Dysphonia as a subtype of Muscle Tension Dysphonia, whereas our framework grouped it with Functional and Psychogenic Dysphonia (Auto A), more closely reflecting the classification proposed by da Silva Moura. Similarly, Reinke’s Edema and Recurrent Paralysis, typically considered distinct, were grouped together by our model (Auto C). These reorganisations led to improved classification accuracy, both within-domain and on external datasets. The full label-to-label correspondence between Auto A/B/C and existing clinical taxonomies is reported in Table 9.

**Table 9:** Correspondence between the data-driven CarLab 2025 groupings and existing clinical taxonomies.

Taxonomy/Category	Functional dysphonia	Hyperfunctional dysphonia	Laryngitis	Leukoplakia	Psychogenic dysphonia	Recurrent paralysis	Reinke’s edema	Vocal Fold Polyp
CarLab 2025								
Auto A	✓	✓	–	–	✓	–	–	–
Auto B	–	–	✓	✓	–	–	–	✓
Auto C	–	–	–	–	–	✓	✓	–
da Silva Moura 2024								
Functional	✓	✓	–	–	✓	–	–	–
Organic	–	–	–	✓	–	✓	–	–
Organofunctional	–	–	✓	–	–	–	✓	✓
USVAC 2025 Level 1								
Functional	✓	–	–	–	✓	–	–	–
Muscle tension	–	✓	–	–	–	–	–	–
Organic	–	–	✓	✓	–	✓	✓	✓
USVAC 2025 Level 2								
Functional dysphonia	✓	–	–	–	✓	–	–	–
Muscle tension	–	✓	–	–	–	–	–	–
Organic inflammatory	–	–	✓	–	–	–	–	–
Organic structural	–	–	–	✓	–	–	✓	✓
Organic neuro muscular	–	–	–	–	–	✓	–	–
Compton 2022								
Inflammatory	–	–	✓	–	–	–	✓	–
Mass lesions	–	–	–	–	–	–	–	✓
Za’im 2023								
Structural dysphonia	–	–	✓	–	–	–	–	✓
Non-Structural dysphonia	–	–	–	✓	–	–	–	–

Each row is a category defined by a particular framework. A checkmark indicates that the disorder is covered under that category; a dash indicates either that the framework does not cover the disorder or that the disorder is grouped under a different category. The table is purely a label-to-label correspondence; we make no claim about the clinical rationale for the data-driven CarLab 2025 partition. Compton 2022 and Za’im 2023 cover only a subset of the SVD diagnoses and therefore have empty cells outside that subset.

This highlights a key clinical insight: while existing diagnostic frameworks are typically grounded in case history and presumed physiological etiology (24), data-driven models are constrained to learn from observable input, in our case the acoustic signal. Although clinicians rely heavily on auditory-perceptual features in practice, these features are believed to correlate only weakly with the acoustic representations captured by signal processing techniques, and thus have not traditionally formed the basis of formal clinical classification frameworks.

By learning from perceptual labels and optimising for acoustic consistency, CarLab 2025 appears to uncover groupings that correspond more closely to how disorders are manifested in the voice signal. These groupings were deemed clinically plausible by our collaborators and generalised well across datasets, suggesting that they capture stable, institution-independent acoustic patterns. A classification framework grounded in such acoustic symptomatology may offer a more reproducible and scalable foundation for future clinical applications, particularly in heterogeneous or low-resource settings.

The clinical-plausibility claim should nevertheless be read as hypothesis-generating, since it rests on commentary from our clinical collaborators rather than on a formal blinded review. A natural follow-up is to ask an independent panel of clinicians, blinded to the data-driven origin of the groupings, to rate each Auto class for clinical coherence. We leave this for a separate study with an appropriately powered clinician panel and inter-rater agreement statistics.

### 4.2 Impact of recording conditions

Our experiments on vocal task variation and data injection revealed that models are highly sensitive to recording conditions and tend to overfit to them. This effect was most clearly demonstrated when injecting only normal samples from a given database: the model’s performance on that specific database collapsed, predicting nearly all samples as normal. This pattern indicates that the model is not learning the underlying vocal characteristics of health vs. disorder, but instead latching onto superficial features tied to recording environments—such as microphone type, room acoustics, or background noise levels—that tend to correlate with class labels in skewed datasets.

Interestingly, although the recording conditions in MEEI were more variable between normal and pathological samples, this overfitting was more severe in Uncommon Voice and VOICED. This suggests that even when using robust SSL-based representations, models still capture—and possibly rely on—dataset-specific recording artifacts, which are particularly problematic in cross-database generalisation.

More encouragingly, injecting just normal samples from additional databases improved classification performance on a held-out dataset (AVFAD), despite that dataset never being seen during training. This indicates that exposure to a broader range of recording environments helps the model decouple pathology from recording artifacts, learning more general patterns in the process.

These findings highlight a critical limitation in current data collection practices. Most clinical voice datasets are recorded in controlled, low-noise environments, which may inadvertently encourage models to exploit environmental regularities rather than pathological cues. To build systems that generalise across institutions and devices, it is essential to diversify the recording conditions in training data.

Although clean, controlled environments have clear advantages and can preserve salient acoustic features useful for differentiation, such features may be easily masked by background noise and thus remain specific to these datasets. Consequently, more work is needed to collect data under variable recording conditions, including differences in background noise, microphone type, recording protocols, and acoustic environments. One potential solution is to encourage patients to record vocal tasks at home, which would inherently introduce greater variability. Future work should explicitly incorporate such diversity to promote models that focus on signal properties reflecting true vocal pathology rather than recording artifacts.

### 4.3 Limitations

Several limitations should be acknowledged. First, our analysis was constrained by the availability of diagnostic labels across databases, with only approximately half of the data from any given database being suitable for analysis due to unclassified labels. Second, our experiments were limited to a single primary training dataset (SVD) for cross-framework comparisons due to insufficient label overlap across databases. Third, the performance gap between binary detection and multi-class classification suggests that current approaches may not fully capture the discriminative features needed for fine-grained classification. Fourth, while data injection improved binary detection performance, these gains did not consistently translate to multi-class classification, indicating that greater data diversity is needed. Fifth, the clinical-plausibility argument for the Auto A/B/C groupings rests on commentary from our clinical collaborators rather than on a formal blinded review. Finally, the exclusion of co-morbidities from our analysis represents a significant limitation, as real-world clinical scenarios often involve multiple concurrent diagnoses.

## 5 Conclusion

In this study, we tested the hypothesis that a data-driven classification framework could improve multi-class classification of vocal pathologies. Our results affirm this hypothesis and demonstrate several key findings.

First, CarLab 2025, derived from model confusion, achieves superior in-domain accuracy compared to established clinical taxonomies, and continues to outperform other taxonomies on multi-class recall in cross-database evaluation.

Second, models trained with structured taxonomies consistently outperformed those trained with narrow, single-disorder labels in out-of-domain settings, highlighting the importance of clinically meaningful groupings for model robustness. Training on a diverse set of vocal tasks also proved more effective for cross-database performance than relying on a single task.

Third, multi-task and multi-criterion training offered no advantage over training a single classifier at the deepest level of granularity. While limited target-domain data injection materially improved binary detection on both injected and held-out databases, this improvement did not extend to multi-class recall, indicating that the binary task is more sensitive to recording-condition exposure than the multi-class task is, and that robust multi-class generalisation will require substantially greater data diversity than two speakers per source database can provide.

In summary, this work validates the utility of data-driven classification and provides a clear, evidence-based path toward developing more robust and generalisable models for vocal pathology detection.

## Data Availability

The data analyzed in this study is subject to the following licenses/restrictions: Voice disorder databases, AVFAD, MEEI, SVD, TORGO, Uncommon Voice, and VOICED are provided on request by their respective authors. Requests to access these datasets should be directed to AVFAD: ieeta-acsa@ua.pt; MEEI: Massachusetts Eye and Ear Infirmary. Voice disorders database, version. 1.03 (cd-rom); SVD: https://stimmdb.coli.uni-saarland.de/; TORGO: https://www.cs.toronto.edu/∼complingweb/data/TORGO/torgo.html; Uncommon Voice: meredith.moore@drake.edu; VOICED: https://physionet.org/content/voiced/1.0.0/.

## Footnotes

^1^https://pypi.org/project/divr-diagnosis.

^2^https://pypi.org/project/divr-diagnosis.
